# Book Review

**Published:** 1934

**Authors:** H. D. Rolleston


### Reviews of Books

**The Physician as Man of Letters, Science and Action.** By T. K. Monro, M.D. Pp. viii., 212. Illustrated. Glasgow: Jackson, Wylie & Co. 1933. Price 10s. 6d.—This is a most interesting little volume, and, indeed, it forms an important contribution to medical history. It consists of biographical notes on the lives and achievements of some four hundred and fifty medical men who distinguished themselves in matters outside professional work. In other words, the author has given us a wonderful gallery of portraits of the poets, authors, soldiers and politicians, of the artists, men of science and philanthropists who have arisen in our ranks. It has taken many years to collect these sketches, but quite recent discoveries and controversies are summarized and inserted. From Bristol and the neighbourhood he draws about a score of names, including Symonds, Thomas Young, the two Beddoes, John Locke and Dover, but we miss J. Cowles Prichard and J. Beddoe as anthropologists, and J. Free the humanist. On the other hand, few Bristolians are alive to the literary claims of Drs. T. Marryat, Jos. Nott, Harington, W. Charleton, and Mortimer Granville, or even know that, besides Smollett, our Bishop Seeker and the pirate Scudamore were qualified medical men, as our author points out. He provides two indices, one of the names of the men and the other of their discoveries or the subjects they worked at. Among many curious entries we may note: "Anæsthesia," the name given to a poor girl whose mother was the first to be delivered under chloroform, and "Vaccinoff," the first Russian child to be vaccinated.

**Lettsom.** By J. J. Abraham. Pp. xx., 498. Illustrated. London: William Heinemann Ltd. 1933. Price 30s.—This is a marvellous picture of the medical world of London in the eighteenth century drawn with great accuracy in every detail like a Dutch painting. Everyone of note plays his part on this stage, for Lettsom knew everyone. Sometimes a fresh actor comes on, and we are told so much about his history and surroundings that Lettsom is forgotten, and one has quite a shock when he re-appears and joins in the fighting, for there always is fighting going on or some reform to be carried out. J. C. Lettsom was born in the West Indies, November, 1744, of Quaker parents. His mother, by the way, had fourteen male children in seven pregnancies, John being one of the last pair. That he was born a Quaker and remained one in spite of their disabilities moulded his whole life. It gave him some powerful friends, such as Fothergill, Franklin and Benjamin Rush; it kept him out of the struggle for honours and offices at Court and from the wild living of the time. Instead of this he concentrated his boundless energies partly on his work, so that he made the largest medical income of that age, and partly on philanthropic movements, prison reform, the Royal Humane Society, the new dispensaries, seaside sanatoria, inoculation and then vaccination, as well as on founding and building up the Medical Society of London, which still survives. The story of each of these movements is given here, supported by many notes and documents of great value. The artistic skill of the writer is well shown in his charming account of the historic dinner where Samuel Johnson, Wilkes and, it seems, Lettsom spent a pleasant hour together by Boswell's scheming. We are told here, too, why the Dillys gave the dinner which, perhaps, Boswell did not know. Again, he gives a terrible picture of the death of old Dr. Fothergill suffering fourteen days' torture from a distended bladder because "no one had the courage to do a suprapubic puncture," and Percival Pott himself could not get in a metal catheter. The book is sumptuously got up, and has one hundred and forty choice illustrations. The only complaint to be made is as to the weight of the volume.

**Inherited Abnormalities of the Skin.** By E. A. COCKAYNE, D.M., F.R.C.P. Pp. x., 394. Illustrated. London: Oxford University Press. 1933. Price 32s.—This book will appeal alike to geneticists and dermatologists, and the author expresses the hope that it may succeed in stiumlating those who deal with affections of the skin to obtain more complete family histories than has hitherto been their custom, and with this object in view he has taken abnormalities of the skin as being superficial and therefore unlikely to be overlooked. The first fifty pages are devoted to the introduction, and contain a clear description of the various types of heredity resulting from different combinations of genes, with simple diagrams to illustrate the Mendelian rules controlling the occurrence of particular characteristics; but the mechanism of Mendelian inheritance is not given in detail, because the reader is expected to be familiar with this part of the subject. The sections dealing with mutations, atavism and stigmata of degeneration will be helpful to workers engaged in the collection and analysis of data, and they warn us of the loose way in which these terms are sometimes used in medical literature; a common example of this is the application of atavism to conditions which are really the result of arrested development, whereas the term should be limited to such conditions as were at one time normal characters of adult man. In the essential part of the work the author has dealt with a great diversity of skin affections, and has taken them in order according to their nature or to the histological structure which they involve; he has analysed a large number of family histories, and has shown very clearly the importance of complete sibships being furnished for determining the kind of heredity in each instance, and he naturally deplores the frequent omission of records of blood relationships so essential in the case of recessives. He hints that with more complete records it might be possible to classify definitely the variants of such diseases as ichthyosis. The references are numerous, giving us an idea of the work involved in compiling this volume and adding to its value as an aid to research. The author has earned a debt of gratitude from the profession for this valuable addition to medical literature, and the publishers are to be congratulated upon the style in which the book has been produced.

**The Enlarged Prostate and Prostatic Obstruction.** Second Edition. By KENNETH M. WALKER, M.B., B.C. Pp. xiii., 223. Illustrated. London: Oxford University Press. 1933. Price 14s.—This book will appeal both to the general practitioner and the surgeon. To the former, in that it gives a lucid and comprehensive description, in all its aspects, of the enlarged prostate and the train of signs and symptoms it produces, culminating in obstruction. Those with which the practitioner is brought in contact are dealt with excellently and the line of action well defined, though perhaps more detail might have been given, particularly for their benefit, in those early cases of enlargement of the prostate which, by suitable treatment, need never arrive at the stage in which operation is indicated. Two most important points to be observed by the man doing general work are wisely emphasized: the danger in the man of blameless life of emptying the bladder, distended as the result of prostatic obstruction, and the vast importance of tracing to its source any urinary hæmorrhage, often associated with the enlarged prostate, without delay, and while the hæmorrhage still exists. Here, also, for the surgeon without much experience in the difficulties and dangers of prostatectomy, will be found an admirable résumé of a wide experience in the handling of these cases, which is among the most difficult of all surgical procedures. Emphasis is laid upon the importance of the preliminary and after-treatment in comparison with which the technique of the operation is but the lesser part. The subject-matter is well up to date, even to a reasoned criticism of the perurethral methods of treatment. The references are adequate, and while some of the illustrations are somewhat crude they are sufficient in their indications.

**Maternal Mortality and Morbidity.** By J. M. MUNRO KERR, M.D. Pp. xviii., 382. Illustrated. Edinburgh: E. & S. Livingstone. 1933. Price 25s.—Dr. Munro Kerr in this book is dealing with a problem about which, as he says, much has been written by different sections of the medical profession. He has approached it along broader lines than is usual, and shows clearly that it is only in this way that any solution to the problem is likely to be found. In addition to this method of approach he deals with the problem in its widest aspect, that is, he includes maternal morbidity, still-births and neo-natal deaths and disability. He has put together a number of facts which are not only useful and interesting, but which should provoke both thought and discussion. Statistics are given clearly in a number of tables and charts, though these are of rather more interest and value to Scotch than to English readers, because many of them are compiled from Scotch figures. The book has brought out the importance of the co-ordination of every service and every agency which has any bearing on the problem of maternal mortality. A drawback to the book is the somewhat unsatisfactory arrangement of the contents of each chapter. The material should be very useful to those who are considering any one section of maternity work and to those who are interested in dealing with these questions as a whole, but it is necessary to go through a great deal of detail before it is possible to appreciate the purport of the chapters. Its value, therefore, as a reference book is not quite as great as it might have been.

**A Short History of Some Common Diseases.** Edited by W. R. BETT. Pp. x., 211. London: Oxford University Press. 1934. Price 10s. 6d.—This is a brilliant collection of papers which describe how our knowledge of each of these diseases grew up. Thus it is not a history of epidemics or of the gradual spread of any disorder, but the story of the gradual building up of the picture of each disease by the discoveries of successive ages. Sir John Fraser in this way traces our idea of tuberculosis from Egyptian and Greek times down to Koch's demonstration of the tubercle bacillus in March, 1882, and onwards. By the way, he fails to mention Bennett of Wrington's early work on the pathology. Sir D'Arcy Power, in all too brief a paper, describes the evolution of our knowledge of syphilis from 1493 down to the discovery of the spirochæte in 1905. Another short article, but of great interest, has to do with the work of Blackall, and still more with that of Richard Bright, on the subject of nephritis. One is impressed throughout the whole volume with the vast amount of historical learning enshrined in these papers, notably in those on the endocrine disorders and epilepsy, where materials for history exist.

**Constitution and Health.** By R. PEARL. Pp. 97. Illustrated. London: Kegan Paul, Trench, Trubner & Co. Ltd. 1933. Price 2s. 6d.—Dr. Raymond Pearl, Professor of Biology in the Johns Hopkins University, has in this short monograph attempted to estimate the importance of the innate constitution of the individual as a causal factor in determining his state of health. Although his language and methods are those of the modern biologist rather than those of the physician, the problem is one of great interest to the medical profession. Dr. Pearl makes no claim to have solved the problem of the correlation between constitution and health, but pleads for the patient accumulation of data, which alone can put the study of constitutional medicine on a scientific basis.

**Bone Growth in Health and Disease.** By H. A. HARRIS, D.Sc., M.B. Pp. xv., 248. Illustrated. London: Oxford University Press. 1933. Price 34s.—Anyone who reads this unique and lucid volume reaps a rich reward. Therein is the mystery of the building of bone made clear, and such tedious processes as the preparatory calcification of cartilage and permanent ossification made an attractive story. The writing is mainly in a thoughtful, scientific vein, though not obscure, and often interspersed with allusions, clinical, historical or zoological. Much of the earlier treatise centres round the author's observations of transverse markings in radiograms of certain young tibiæ. These, he shows, are legacies of spells of arrested growth during early life. By the changing position of these dense lines in repeated radiograms over long periods the opinion formed by earlier experimenters is confirmed, namely that length is added to bones not by interstitial increase, but by growth at the epiphyseal line. In later chapters he demonstrates clearly how the vulnerable site of bone deposition is affected by forces of disorder and disease. He spares no trouble to clarify the various characteristic reactions of bone to different endocrine, metabolic, toxic and infective influences, and shows them to be all modifications of one simple system, the "annulus of mitosis." The diagram illustrating this on page 146 may be regarded as the key picture to the author's conception of the subject. Antenatal and senescent aspects are included in the consideration of the growth of bone and its forerunner, cartilage. In style, biological range, restraint and regard for truth this work is worthy of the classics of English medicine. The illustrations and print are of the fine order appropriate for such masterly matter, which should form the foundation of his study for every postgraduate student of bone surgery.

**This Panel Business.** By G. P. Pp. xii., 364. London: John Bale, Sons & Danielsson Ltd. 1933. Price 10s. 6d.—This book gives a good general survey of panel practice both in this country and abroad. It enters fully into its future. The author criticizes fearlessly approved societies, insurance committees, lay newspapers, the Ministry of Health, panel committees, the Insurance Acts Committee, and doctors themselves. He points out serious difficulties in the way of public medical services. He makes out a very good case for the restoration of the cuts in the pay of panel practitioners, as this does, in fact, include his professional expenses as well as his profits. He ends with valuable suggestions for the future of panel practice; these should be very carefully studied by all panel doctors. They will find much helpful information in this volume. The book is well written and readable. There are one or two minor inaccuracies which do not affect its value, *e.g.* the author comments on the lack of lectures to students on insurance matters: such lectures are given.

**Recent Progress in Medicine and Surgery.** Edited by Sir John Collie, C.M.G., M.D. Pp. xii., 368. Illustrated. London: H. K. Lewis & Co. Ltd. 1933. Price 16s.—There have been many books published in the past two or three years dealing with recent advances in some particular branch of medicine or surgery, but Sir John Collie's volume is unique in that it aims to cover the additions to the whole field of medicine and surgery in the period from the end of the war to the present time. Twenty-one different authors contribute, and the articles are almost uniformly of the greatest practical value, full of information and eminently readable. This is a book to which the busy practitioner will make frequent reference, for the question of recent progress in treatment has been put in the foreground throughout. Another very important function fulfilled by this work is that it clearly and definitely defines the position of medical knowledge at the present time in many controversial matters. In this connection we may mention the contributions on endocrinology, neurology, bacteriology, radiology and vitamins. The author of each has largely eliminated all that is conjectural and problematical, and has brought into prominence the established facts which are of practical value in regard to both diagnosis and treatment.

**Rheumatism in General Practice.** By M. B. Ray, M.D. Pp. x., 404. Illustrated. London: H. K. Lewis & Co. Ltd. 1934. Price 16s.—Lord Horder, in his foreword, calls this book "the best account of rheumatism in our language." Not having read all the books written on the subject, we cannot confirm his rather sweeping statement! Nevertheless, it is an excellent book, and as useful to the specialist as to the general practitioner, for whom, according to the title, it is intended. Its scope is rightly large, "rheumatism" covering a multitude of diseases, but the classification and diagnosis of the various types is usually sound. The old conceptions of the ætiology of rheumatic diseases is greatly modified and put on a firmer and more practical basis. Physiotherapy is not as neglected as usual, though it is apt to be relegated to the might-be-trieds; after all, the practitioner *knows* what "may be tried," the important thing to appreciate is what is likely to cure his patient. It is surprising to find repeated the old erroneous idea that "heat is driven in" (when it is actually generated) by diathermy. Perhaps the most useful sections in the book are the anatomical notes, which, though somewhat

F

crowded, are splendid refreshers to one's overburdened memory. The whole book would have been improved by more illustrations, for apart from some half-dozen X-ray plates of hands (excellent as far as they go) there are only a couple of diagrams. The printing on the whole is good, and the heavy-typed headings are excellent for reference. The index is nothing like adequate, and the binding is not too good for the weight of the book, to which a thirty-two page catalogue adds bulk but not elegance.

**Heart Disease.** By CRIGHTON BRAMWELL, M.D. Pp. vii., 244. Illustrated. London: Edward Arnold & Co. 1932. Price 12s. 6d.—This is an excellent little book both for the student and the practitioner. Within the compass of a little more than two hundred pages the author portrays in a clear and lucid manner what might be termed the essentials of heart disease. Diagrams and radiograms are inserted where required, while electrocardiograms are clearly described throughout. The term valvular lesion is preferred to valvular disease, but in his endeavour to impress upon the student the modern conception of the all-important state of the myocardium, the author dismisses valvular lesions without making clear the difference between hæmic and organic murmurs. Again, a little less sketchiness in describing congenital heart disease would prove to be of great value in a work which is written primarily for the student. The chapters on angina pectoris and coronary occlusion are perhaps outstanding as concise and masterly descriptions of these conditions. With the few exceptions above mentioned, the pages of this book are vibrant with the voice of one who speaks clearly and authoritatively.

**Therapeutic Uses of Infra-Red Rays.** Second edition. By W. A. TROUP, M.C., M.B. Pp. xiv., 92. Illustrated. London: The Actinic Press. 1933. Price 6s. 6d.—The first edition has been revised and the physical side brought up to date. The book should appeal to general practitioners, who will find the technique and apparatus required for the treatment by the infra-red rays simply and clearly described. These are most useful for the relief of pain and cure of many of the common painful ailments which one meets in general practice, especially in those conditions where the absorption of infiltrations and exudations are required. The latter part of the book is devoted to the clinical conditions in which

Dr. Troup has found infra-red radiation of value, and is a guide to practitioners who desire to add this method of treatment to their therapeutics. The book ends with an interesting appendix on photography by infra-red radiation.

**Clinical Contraception.** By GLADYS M. COX, M.B., B.S. Pp. ix., 173. Illustrated. London: William Heinemann Ltd. 1933. Price 7s. 6d.—This is a most comprehensive book, dealing in detail with the clinical methods of contraception. The pros and cons of each method are clearly indicated and the choice of method, suitable to various types of patient, given. The various contraceptive appliances are described and illustrated, and their correct situation in the genital tract is shown by diagrams. The whole is well written and is an up-to-date summary of our present knowledge of the subject and can be especially recommended as a practical treatise for the general practitioner, the gynæcologist and those in charge of birth control clinics.

**The Hygiene of Marriage.** Fourth edition. By ISABEL E. HUTTON, M.D. Pp. x., 146. Illustrated. London: William Heinemann Ltd. 1933. Price 5s.—This, the fourth edition of the book, maintains the high standard of previous editions. It has been amplified and the chapters on birth control and contraceptives brought up to date. The added chapter on "Childlessness" is to be welcomed, because, as the author states, this condition can often be remedied as the result of medical advice. The book can be recommended to those who are about to be married as a wholesome one, and will be useful to those practitioners whose advice on this subject may be sought.

**Sex Efficiency Through Exercise.** By TH. H. VAN DE VELDE, M.D. Pp. xvii., 163. Illustrated. London: William Heinemann Ltd. 1933. Price 25s.—It is interesting that "Good Taste" is a phrase far more frequently applied in a social or æsthetic signification than to the actual physical titillation of the palate. This book bears a striking resemblance to the curate's egg. The parts which are excellent are devoted to detailed descriptions of exercises for women, mainly for use during pregnancy and the puerperium. Their value is enhanced by a well-chosen series of ingenious "flicker" pictures, which by a kinematograph effect show clearly just how each exercise should be performed. In the remaining smaller portion is described how exercises of the abdominal and pelvic muscles can be applied to increase physical satisfaction in coitus. Certain oriental races, we believe, attach much importance to this subject—and actually prefer their eggs rotten. We feel that it is not mere insular prejudice that leads us to emulate the curate's reticence.

**An Outline of Practical Obstetrics for Nurses.** By R. S. S. Statham, M.D., Ch.M. Pp. 139. Bristol: John Wright & Sons Ltd. 1933. Price 2s. 6d.—The family of midwifery booklets is large, but this youngest addition is welcome. For though the family likeness is undeniable, there is that "something different" which makes it attractive. The attraction is not based on its illustrations, for it has none. The preface supplies the clue. The little book is the achievement of a clear-cut objective. It covers the syllabus for the Central Midwives Board Examination, and whilst admittedly designed to facilitate revisional preparation for that examination with its steadily rising pass-standard, it rigorously avoids cram lists throughout. The comparative table of mechanisms on the large end fly-leaf is well drawn for easy reference. The author may be well assured that while, as he surmises in the preface, it certainly will "while away some of the long hours of waiting on the District," the book, with its clear captions and fifteen pages of well-typed index, also lends itself to ready reference in stressful times. The contents of the sixteen chapters are best adapted to the study of those having knowledge and experience, such as nurses possess, and the subjects of dominant importance, such as ante-natal care, puerperal infections, hæmorrhage and infant-feeding are adequately and very helpfully dealt with. The booklet is a mine of well-arranged information within, and in form and size a handy vade-mecum.

**Chronic Nasal Sinusitis and its Relation to General Medicine.** Second edition. By P. Watson-Williams. Pp. xx., 262. Illustrated. Bristol: John Wiight & Sons Ltd. 1933. Price 15s.—Specialism, the necessary outcome of the vast expansion of the science and practice of the healing art, and moreover now a powerful agent in the progressive advance of medicine, had to suffer the displeasure of the orthodox leaders of the profession during a considerable part of the last century. Many now living can remember when its existence was little more than tolerated by those in the seats of the mighty, and that to be on the staff of a special hospital was not a recommendation for election on to that of the great teaching hospitals in London. It was said that specialism tended to a narrow outlook, and by focusing attention on the trees obscured the view of the wood. This may have had some truth, and that some enthusiasts were not exempt from the human liability to error is hardly surprising. However this may have been, the position of legitimate specialism has long been established, and no better example of its value need be quoted than this work of Dr. Patrick Watson-Williams, which three years after its appearance has now passed into a deserved second edition. This generously-illustrated volume appeals widely to medical men for its account of systemic infection, as well as to specialists, who will find much to interest them in the five chapters devoted to the detailed consideration of the diagnostic methods and treatment of sinusitis. The conception of focal sepsis, which William Hunter has set out in many contributions since 1889, followed by the late Frank Billings of Chicago, is traced back by the author to a much earlier date; for example, the astrologist and quack Nicholas Culpeper and the Swiss physician J. J. Wepfer in the seventeenth century had some such inkling. The nasal sinuses are specially suitable for the study of focal sepsis, for they are normally sterile, and are readily accessible to bacteriological investigation, and thus any infection can be determined. It is pointed out that although the primary sites of focal infection vary, the distribution of systemic toxæmia and hæmic secondary infections does not depend on any special source of origin; on the other hand, there are some obvious differences in the local spread of infection determined by anatomical connections. A specially interesting chapter deals with secondary infections of the orbit and eye, a subject the author began to elucidate in 1892. A new feature in this edition is the discussion of some problems in chronic sepsis, such as the nature of agranulo-cytosis, mixed infections, leontiasis, and other obscure diseases of bone. Each chapter has a bibliography, and the volume is piously dedicated to the Bristol Medical School in the year of its centenary as a tribute from one of her sons.

H. D. ROLLESTON.

